# MINDFULNESS PRACTICE IN OLDER ADULTS: MIXED-METHOD FINDINGS FROM AN OUTPATIENT GROUP INTERVENTION

**DOI:** 10.1093/geroni/igae098.4240

**Published:** 2024-12-31

**Authors:** Christopher Griffith, Jonathan Sundby, Tiana Broen, Leilani Feliciano

**Affiliations:** University of Colorado Colorado Springs, Colorado Springs, Colorado, United States; University of Colorado Colorado Springs, Colorado Springs, Colorado, United States; University of Colorado Colorado Springs, Colorado Springs, Colorado, United States; University of Colorado Colorado Springs, Colorado Springs, Colorado, United States

## Abstract

Mindfulness can be thought of as a skill that centers on directing attention, in the present moment, with non-judgment. Mindfulness skills are effective strategies for addressing psychological symptoms, including improving mood, stress, chronic pain, sleep, and well-being in older adults. To further examine the effects of mindfulness practice in a group setting, two 8-week mindfulness skills building groups were conducted at a community outpatient clinic for older adults in Southern Colorado. Participants (n = 11) completed pre-post measures on depression, anxiety, stress, well-being, pain, sleep, and trait-mindfulness. The final session was recorded and transcribed to allow for thematic analysis. During this session, participants were asked how mindfulness practice influenced them and what changes they may have noticed. Some responses included themes related to outcome measures (e.g., stress, well-being), with additional themes emerging related to: 1) overall effectiveness, 2) greater insight, 3) interpersonal functioning, 4) group cohesion, 5) context, and 6) filler. Paired-samples t-tests were conducted demonstrating significant improvements in depressive symptoms [t(5) = 4.84, p <.01], anxiety [t(8) = 2.79, p <.05], perceived stress [t(9) = 3.03, p <.05], well-being [t(8) = -3.90, p <.01], and trait mindfulness [t(9) = -6.01, p <.001]. Study findings support mindfulness for symptom reduction and improved mental health and well-being in older adults, and provide a better understanding of subjective benefits to participating in mindfulness groups. Thematic results indicated that older adults value mindfulness practice and appreciate the shared experience of learning and practicing with their peers.

